# Exercise physiology with a left ventricular assist device: Analysis of heart-pump interaction with a computational simulator

**DOI:** 10.1371/journal.pone.0181879

**Published:** 2017-07-24

**Authors:** Libera Fresiello, Frank Rademakers, Piet Claus, Gianfranco Ferrari, Arianna Di Molfetta, Bart Meyns

**Affiliations:** 1 KU Leuven, Department of Cardiac Surgery, Leuven, Belgium; 2 Institute of Clinical Physiology, National Research Council, Pisa, Italy; 3 KU Leuven, Department of Cardiovascular Sciences, Leuven, Belgium; 4 Nałecz Institute of Biocybernetics and Biomedical Engineering, Polish Academy of Sciences, Warsaw, Poland; 5 Medical and Surgical Department of Pediatric Cardiology, Pediatric Hospital Bambino Gesù, Rome, Italy; Scuola Superiore Sant'Anna, ITALY

## Abstract

Patients with a Ventricular Assist Device (VAD) are hemodynamically stable but show an impaired exercise capacity. Aim of this work is to identify and to describe the limiting factors of exercise physiology with a VAD. We searched for data concerning exercise in heart failure condition and after VAD implantation from the literature. Data were analyzed by using a cardiorespiratory simulator that worked as a collector of inputs coming from different papers. As a preliminary step the simulator was used to reproduce the evolution of hemodynamics from rest to peak exercise (ergometer cycling) in heart failure condition. Results evidence an increase of cardiac output of +2.8 l/min and a heart rate increase to 67% of the expected value. Then, we simulated the effect of a continuous-flow VAD at both rest and exercise. Total cardiac output increases of +3.0 l/min (+0.9 l/min due to the VAD and +2.1 l/min to the native ventricle). Since the left ventricle works in a non-linear portion of the diastolic stiffness line, we observed a consistent increase of pulmonary capillary wedge pressure (from 14 to 20 mmHg) for a relatively small increase of end-diastolic volume (from 182 to 189 cm^3^). We finally increased VAD speed during exercise to the maximum possible value and we observed a reduction of wedge pressure (-4.5 mmHg), a slight improvement of cardiac output (8.0 l/min) and a complete unloading of the native ventricle. The VAD can assure a proper hemodynamics at rest, but provides an insufficient unloading of the left ventricle and does not prevent wedge pressure from rising during exercise. Neither the VAD provides major benefits during exercise in terms of total cardiac output, which increases to a similar extend to an unassisted heart failure condition. VAD speed modulation can contribute to better unload the ventricle but the maximal flow reachable with the current devices is below the cardiac output observed in a healthy heart.

## Introduction

Exercise incompetence is a hallmark of heart failure and is the result of multiple interacting cardiac and vascular impairments. In end-stage heart failure, medication is no longer adequate and heart transplantation or a mechanical assist device (VAD) are the only available treatment options besides palliation. VAD patients, although hemodynamically stable at rest, reach only 50% of the expected exercise capacity [1]. Several clinical studies investigated why VAD therapy cannot restore exercise capacity to normal values [2,3]. What emerged so far is that VAD patients show similar limitations as heart failure patients such as autonomic nervous system impairment, chronotropic incompetence, poor perfusion of exercising muscles, right ventricular dysfunction and iron deficiency [4]. Heart failure and VAD patients show also similar hemodynamics from rest to exercise, particularly in terms of wedge pressure, pulmonary pressure and right atrial pressure elevation [5]. In addition, cardiac output increase is limited despite the contribution of the VAD. Some studies investigated if a VAD speed modulation during exercise could overcome this but results showed little effects on exercise performance [6] and on cardiac output [5].

These studies evidence the complexity of the exercise pathophysiology in VAD patients and raise some questions about the interplay between the native ventricle and the VAD during exercise induced increases in cardiac output.

Given these premises, this study is intended to provide a systematic overview of the knowledge on evolution of VAD patients’ hemodynamics during exercise. At present there are only a few studies describing exercise pathophysiology with a VAD [2,3,7], and even less reporting both systemic and central hemodynamics [5]. We searched and grouped the data available in the literature, and analyzed them using a cardiorespiratory simulator. The latter is based on a computational model of the cardiovascular and respiratory systems [8] that was adapted to reproduce the pathophysiology of severe heart failure with a VAD at rest and at peak exercise. The simulator allowed to investigate the interaction of the cardiovascular system and in particular of the native heart with a VAD, providing highlights on how hemodynamics evolves during exercise and why the VAD can only partially improve it.

## Materials and methods

### Model structure

We developed a lumped parameters cardiorespiratory simulator in LabVIEW 2014 (National Instrument, Austin, TX, USA) able to reproduce exercise in different pathophysiological conditions.

The left and right hearts are represented by a time-varying elastance model [9], the systemic and pulmonary circulations are represented by Windkessel models arranged in series and/or in parallel [10].

**Fig 1:**
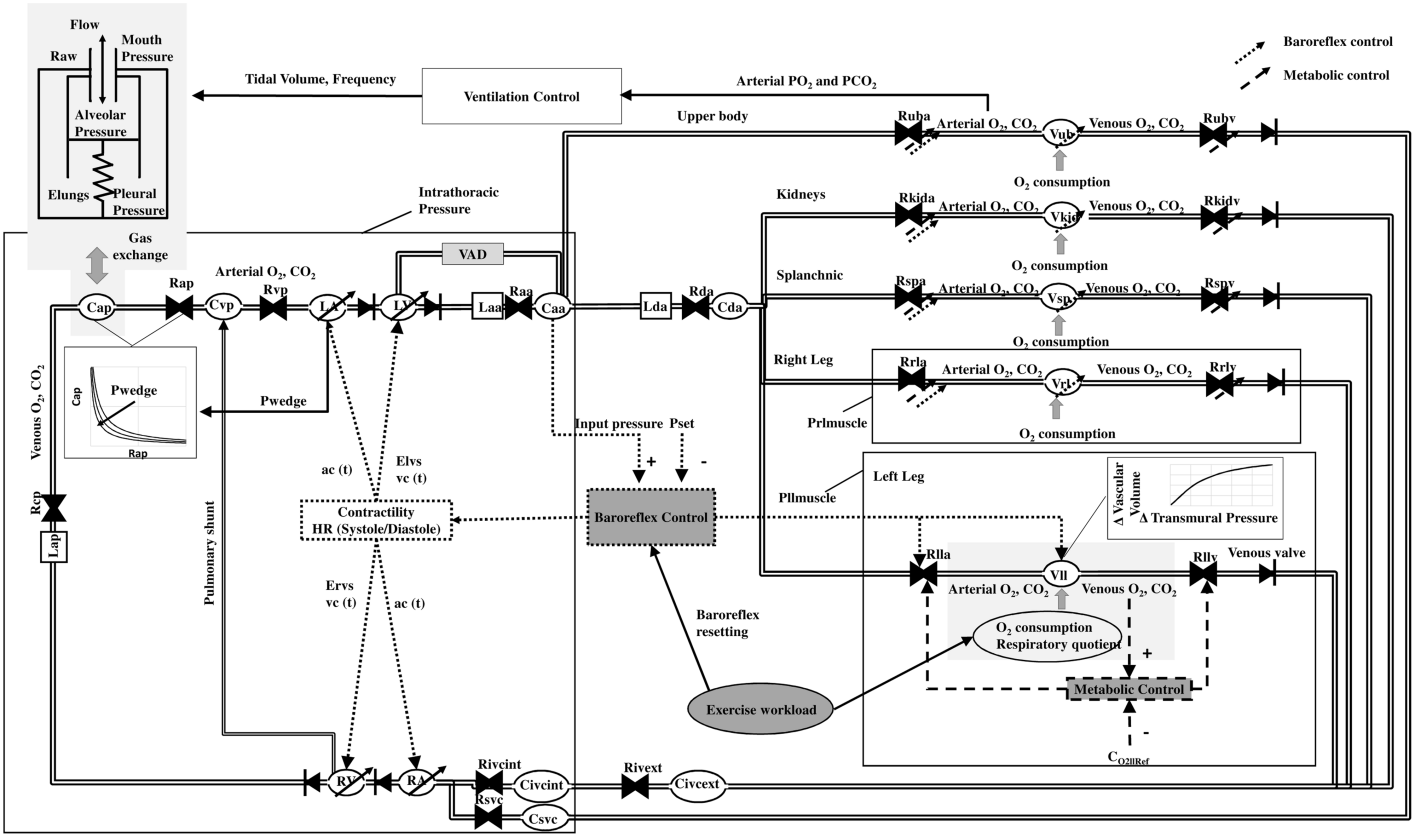
Simulator overview. Block diagram of the cardiorespiratory system and its connection with a VAD. The left and right atria (LA, RA) are represented as linear compliances contracting during diastole (S1eq. 1, S1 eq. 2), left and right ventricles (LV, RV) are represented with a time–varying elastance model during systole (S1 eq. 3, Eq 1) and as a non-linear compliances during diastole (Eq 2). Ascending and descending aorta are represented with a resistance, inertance and compliance (Laa, Raa, Caa and Lda, Rda and Cda). Every i^th^ circulatory district is represented by an arterial resistance (Ria), a compliance (Ci) and a venous resistance (Riv). For the upper body, splanchnic circulation, legs and inferior vena cava the compliance is non-linear for high values of transmural pressure (S1 eq. 6). The venous resistance of left and right legs are represented as starling resistors, under the effect of muscular pressure (Prlmuscle and Pllmuscle). The pulmonary arterial circulation is represented by an inertance (Lap), a characteristic resistance (Rcp), a compliance (Cap) and a resistance (Rap). Cap value is a function of Ras and of Pwedge as described in S1 eq. 7. Pulmonary venous circulation is represented with a resistance (Rpv) and a compliance (Cpv). The VAD is simulated between the left ventricle and the aorta according to S1 eq. 7. A detailed overview of the gas exchange in the tissues and of the peripheral controls implemented in the models is reported for the left leg circulation as an example. The same structure is applied to upper body, kidneys, splanchnic circulation and right leg. Lungs are represented as one compartment where blood is oxygenated and decarbonized. Ventilation mechanics is represented starting from the pleural pressure (whose value depends on the tidal volume and the ventilation frequency). Then, a lung elastance (Elungs) and a resistance of the airways (Raw) permits to calculate alveolar pressure and flow.

The simulator also includes the baroreflex and metabolic peripheral controls, ventilation control and mechanics, gas exchange in lungs and tissues. A complete description of the model is provided in [8]. A schematic representation of the model structure is provided in Fig 1 for clarity.

The simulator is fed with hemodynamic and respiratory parameters at rest. Exercise is then simulated by increasing the oxygen consumption in the body, differentiating between exercising and non-exercising regions. Since the simulator does not include metabolic activity at cellular and tissue levels, the oxygen consumption and the respiratory quotient are actually two inputs of the simulator. Their values were taken from the literature and refer to HF patients characterized by a lower muscular oxygen extraction and an earlier anaerobic metabolism (see [8] for more details). Given the local oxygen consumption level and the respiratory quotient, the simulator calculates the concentration and partial pressures of oxygen and of carbon dioxide in each vascular compartment (upper body, kidneys, splanchnic circulation, legs).

These local oxygen concentrations are used by the metabolic control to induce vasodilation locally in where oxygen consumption increases. In addition, the baroreflex control contributes to the regulation of peripheral resistances. The baroreflex control is generated centrally, from the pressure signal in the aortic arch, and it regulates heart rate, peripheral arterial resistance, venous tone and ventricular contractility. A model of “baroreflex resetting” is also included, so that during exercise the vagal nerve withdraws and the sympathetic nerve is overstimulated. As a result, a positive chronotropic and inotropic effect on the heart is observed.

Mechanical ventilation is reproduced in terms of lung volume, pleural pressure and intrathoracic pressure (the latter affecting the vessels inside the chest). Given the oxygen and carbon dioxide partial pressures in the upper body, a dedicated control calculates the minute ventilation. For increasing levels of workload, the minute ventilation augments with consequent increase of tidal volume and of ventilation frequency.

The ventilated air is used in the gas exchange module at the level of the pulmonary circulation where the oxygenation and decarbonization of blood takes place.

The simulator, arranged with all the components mentioned above, was already used to reproduce exercise physiology in healthy and in heart failure conditions and it was validated with data from literature [8].

In the present work we further adapted the simulator to reproduce the hemodynamic physiology of exercise in severe heart failure condition, with and without a VAD. To this aim, some changes/improvements were implemented:

- The systole/diastole duration ratio changes according to the heart rate in such a way that diastole shortens more for a high heart rate and vice versa,
- A model of atrial contraction was implemented to reproduce the contribution of atrial chambers to fill the ventricles during late diastole
- Venous vessels are represented as collapsible tubes whose pressure-volume relationship becomes nonlinear for high values of transmural pressure
- The effect of the increase of pulmonary venous capillary wedge pressure on the pulmonary vasculature was implemented
- A VAD model based on the pressure-flow characteristics of HeartMate II (Thoratec Corporation) was added between the left ventricle and the ascending aorta of the simulator

More details about how these improvements were implemented are reported in S1.

### Simulator parameters

Parameters such as resistances, compliances, ventricular contractility represent the inputs of the simulator. The outputs of the simulator are: pressure, flow, volume, O2 and CO2 concentration profiles in every section of the cardiovascular system.

A detailed description of the procedure for the determination of the input parameters was provided in [8]. Here we only provide those parameters which required a new characterization in order to produce a more severe heart failure (HF) than the one simulated in [8] and the effect of a VAD (HF+VAD). The complete list of parameters is reported in Table 1 and how they were estimated is explained in the next paragraphs.

**Table 1:** Simulator parameters.

Symbol	Parameter	Unit	HF	HF+VAD
***HR***	Heart Rate	bpm	81	76
***al/ar*** ***bl/br***	Left/Right ventricular filling	mmHg cm^-3^	0.0532/0.050 0.0194/0.045	0.01/0.044 0.0194/0.045
***Elvs/Ervs***	Left/right ventricular end-systolic elastance	mmHg/cm^3^	0.5/0.37	0.5/0.37
***Ruba***	Upper body arterial resistance	mmHg·s/cm^3^	5.2	4.2
***Rkida***	Kidney arterial resistance	mmHg·s/cm^3^	5.4	4.3
***Rspa***	Splanchnic arterial resistance	mmHg·s/cm^3^	4.0	3.2
***Rlla/Rrla***	Left/Right leg arterial resistance	mmHg·s/cm^3^	9.4/9.4	7.6/7.6
***Rap***	Pulmonary arterial resistance	mmHg·s/cm^3^	0.18	0.10
***C***_***O2ubvRef***_	Reference venous oxygen concentration in the upper body	ml O_2_/dl blood	10	10
***C***_***O2kidvRef***_	Reference venous oxygen concentration in the kidneys	ml O_2_/dl blood	13.5	13.5
***C***_***O2spvRef***_	Reference venous oxygen concentration in the splanchnic circulation	ml O_2_/dl blood	11	11
***C***_***O2llvRef***_***/C***_***O2rlvRef***_	Reference venous oxygen concentration in the left/right leg	ml O_2_/dl blood	10	10
***Pset***	Set-point pressure baroreflex	mmHg	88	91
***Vlv***_***0***_***/Vrv***_***0***_	Left/right ventricular zero pressure volume	cm^3^	50/-20	0/-20

List of parameters used to characterize the simulator in HF and in VAD rest conditions.

#### Heart parameters

We here describe how the systolic and diastolic properties of the ventricle were represented.

The contractility of both the left and right ventricles was implemented by a time-varying elastance model [9]:

Plv(t)=Elvs ⋅ vc(t) ⋅ (Vlv(t)−V0)+Pintr(t) (1)

Where *Plv* is the left ventricular pressure, *Pintr* is the intrathoracic pressure, *Vlv* is the left ventricular volume, *V0* is the zero pressure filling volume, *t* is time ranging from 0 s to 60/HR s, *Elvs* is the end-systolic elastance and *vc* is an adimensional ventricular contraction function ranging from 0 during diastole to 1 ad end systole (see the appendix for more information). For the right ventricle a similar equation was implemented.

To simulate the impairment of the systolic function, typical for HF, *Elvs* was reduced to 0.5 mmHg/cm^3^ and *V0* was set to 50 cm^3^. For HF+VAD we used the same value for *Elvs* but *V0* was reduced to 0 cm^3^ to account for the unloading effect of the VAD on the left ventricle. For the right ventricle a similar equation was implemented. Right ventricular contractility (*Ervs*) was taken from [11] and refers to idiopathic dilated cardiomyopathy patients.

The left ventricular stiffness was represented with an exponential function describing the relation between pressures and volumes:

$$Plv\left(t\right)=al\;\cdot \;e^{bl\cdot Vlv\left(t\right)}+Pintr\left(t\right)$$ (2)

Where *al* and *bl* are constant parameters describing the stiffness properties of the ventricle during diastole. For HF, *al* and *bl* were taken from [12] and refer to end-diastolic pressure-volume relationship of ex vivo human hearts with dilated cardiomyopathy.

**Fig 2:**
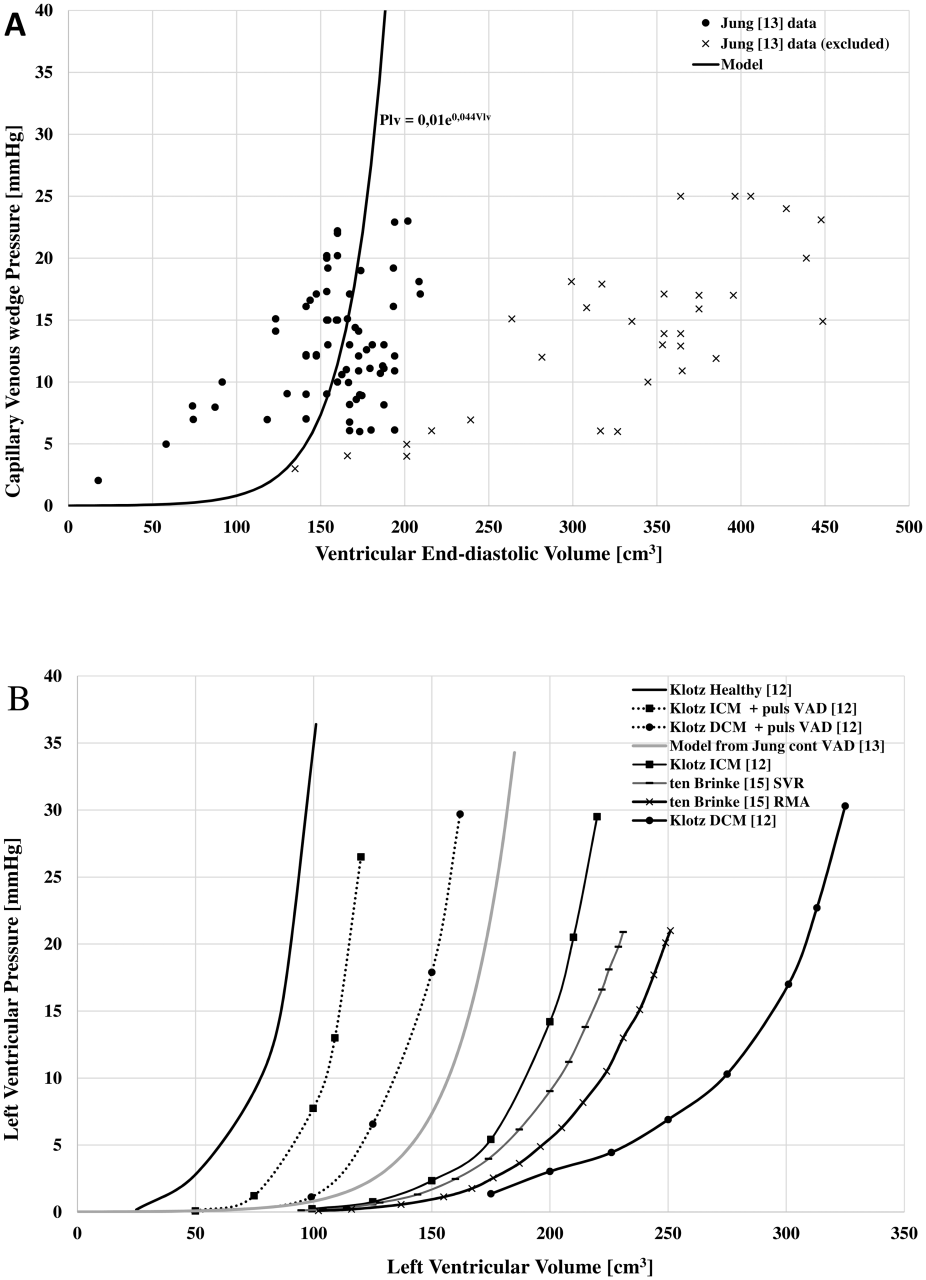
Left ventricular diastolic characteristic. (A) Ventricular stiffness in patient with a continuous-flow VAD. The continuous line indicates the exponential function that was obtained by fitting the data (●) taken from Jung et al [13]. Symbol (x) indicates data excluded from the analysis. (B): Comparison between the ventricular stiffness calculated in panel (A) and the stiffness in other pathophysiological conditions taken from literature [12,13,15]. From left to right curves refer to patients with: healthy condition, ischemic cardiomyopathy and a pulsatile VAD, dilated cardiomyopathy and a pulsatile VAD, continuous-flow VAD, ischemic cardiomyopathy, scheduled for ventricular restoration, mitral regurgitation, dilated cardiomyopathy.

For HF+VAD, parameters *al* and *bl* were estimated fitting the data of 10 patients with a continuous-flow assist device [13]: data reflect the capillary venous wedge pressure and the end-diastolic ventricular volume estimated from the end-diastolic diameter using the Teicholz formula (see Fig 2-A). An exponential curve fitting was used to estimate the diastolic stiffness from these data as shown in in Fig 2-A.

Three patients were excluded from the analysis since they had a very different stiffness in comparison to the rest of the group. This difference might be due to large variety of VAD therapy duration (668 ± 596 days), so that in some patients it is likely to assume that the VAD was not left in place for sufficient time to reduce left ventricular dilatation [14].

#### Circulation parameters

Systemic and pulmonary vascular resistances were estimated from literature data relative to rest condition as follows:

Ras=(Pas−Pcv)/CO (3)

Rap=(Pap−Pwedge)/CO (4)

Where *Ras* (*Rap*) is the systemic (pulmonary) arterial resistance, *Pas* is the mean systemic arterial pressure, *Pcv* is the mean central venous pressure, *Pap* is the mean pulmonary arterial pressure, *Pwedge* is the pulmonary capillary wedge pressure and *CO* is the total cardiac output.

*Ras* and *Rap* in HF were calculated averaging data from [3,16,17,18]. For HF+VAD *Ras* was calculated considering data from [3,5,13], *Rap* averaging data from [3,5]. *Ras* was then split into the resistance of upper body, kidneys, splanchnic circulation and lower limbs as reported in [8].

#### Controls parameters

Two main controls are implemented for the vasculature: the metabolic and the baroreflex controls. We will describe them here briefly.

Computationally, the metabolic peripheral control regulates vasodilation in the circulatory districts regions where oxygen consumption increases. This control works around a reference value of the venous oxygen concentration, each i^th^ vascular region has a specific reference value (*C*_*O2iRef*_). If the actual venous oxygen content falls below *C*_*O2iRef*_, hypoxic vasodilation occurs locally. Heart failure patients are chronically exposed to low values of perfusion, therefore their tissues are used to lower values of oxygen concentration. To take this phenomenon into account we set a lower *C*_*O2iRef*_ for HF and for HF+VAD in all the vascular compartments (see Table 1).

The baroreflex control works around a set-point value (*Pset*): if the actual aortic pressure is lower than *Pset* vasoconstriction occurs, heart rate and contractility increase and venous tone reduces and vice versa. From a physiological point of view, *Pset* represents the equilibrium value and it was characterized in the simulator at rest condition: for HF we averaged the mean systemic pressure at rest taken from [3,16,17,18], for HF+VAD we averaged the data of [3,5,13]. During exercise *Pset* automatically increases due to the “baroreflex resetting phenomenon” that progressively shifts the baroreflex control towards higher pressure levels.

We also introduced a sympathovagal imbalance, by re-adjusting the sympathetic and vagal nerve controls on HR. This permits to simulate the chronotropic incompetence observed during HF. More details about the controls mechanisms, their equations and parameters values are reported in [8].

Concerning the pulmonary vasculature, the hemodynamic data of HF patients show a slight decrease of *Rap* from rest to exercise (from 0.183 to 0.151 mmHg·s/cm^3^) [3,16,17,18]. We therefore decided not to implement any hypoxic vasoconstriction mechanism for the pulmonary vasculature. The simulator was therefore fed with the *Rap* at rest and this value was kept constant during exercise simulations.

### Simulation procedure

Two simulation sets were performed: one for the HF and the other for the HF+VAD.

To simulate exercise in HF we started by characterizing the simulator at rest. For this purpose we fed hemodynamic and respiratory parameter values at rest into the simulator as explained in the previous paragraphs. The simulator was left running until a steady condition was reached and data were collected. Then we increased oxygen consumption up to 11.5 ml/min/kg (bicycle workload of 56 watts) which is the peak level for severe HF according to [3,18]. The simulator was left running until a steady condition of peak exercise was reached and data were collected again.

The same procedure was repeated for HF+VAD. We first set the VAD speed at 9500 rpm and collected the data at rest condition. Then we increased oxygen uptake up to 15.2 ml/min/kg (bicycle workload of 80 watts) which is the peak level according to [19]. The simulator reached the steady condition and data were collected again. We then increased VAD speed to 12000 rpm at peak exercise and the last set of data was collected.

Simulation results refer to pressures and flows in the cardiorespiratory simulator, reported as average values, calculated over 15 heart cycles to reduce the ventilation effect. As pulmonary capillary wedge pressure, we considered the average value of left atrial pressure obtained from the simulator.

In addition, continuous pressure and volume data of the left ventricle were recorded.

## Results

Fig 2-A shows the diastolic pressure-volume data of the left ventricle in 10 patients with a HeartMate II pump [13]. The continuous line is the ventricular stiffness estimated from this data using an exponential fitting procedure.

In Fig 2-B we compare the curve estimated in Fig 2-A with the ventricular stiffness of other pathophysiological conditions. These refer to ex vivo human hearts with ischemic and dilated cardiomyopathy before and after the implantation of a pulsatile-flow VAD (TCI HeartMate, Thoratec, Pleasanton, CA [12], and patients with heart failure (New York Heart Association (NYHA) class III and IV) scheduled for restrictive mitral annuloplasty (RMA) and/or surgical ventricular restoration (SVR) [15].

**Fig 3:**
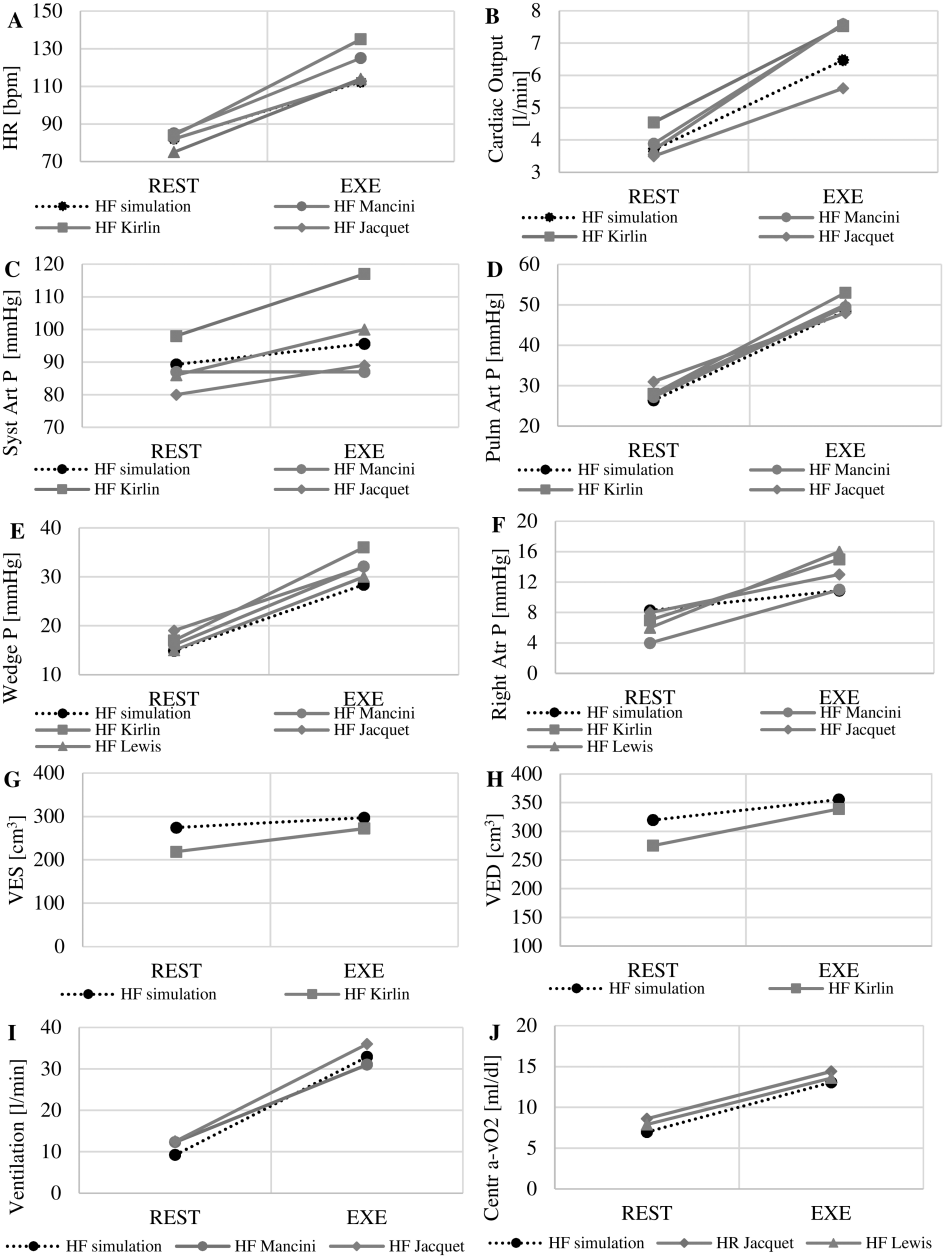
Comparison between literature and simulation studies at rest (REST) and at peak exercise (EXE) in HF condition. Data refer to: heart rate (A), cardiac output (B), systemic arterial pressure (C), pulmonary arterial pressure (D), pulmonary capillary wedge pressure (E), right atrial pressure (F), left ventricular end systolic volume (G) and end diastolic volume (H), ventilation (I), central arteriovenous oxygen difference (J).

Fig 3 shows the comparison between literature data and simulations results for HF conditions at rest and at peak exercise. The literature data summarized in Fig 3-A refer to HF patients with an average age of 53 years. This corresponds to an expected peak HR at exercise of 220–53 = 167 bpm. Due to the sympathovagal imbalance implemented in the simulator, the peak HR reached for HF is 112 bpm, corresponding to 67% of the expected value. Cardiac output increases from 3.7 to 6.5 l/min. Pressures also increase: mean systemic arterial pressure from 89 to 95 mmHg, mean pulmonary arterial pressure from 26 to 48 mmHg, wedge pressure from 14 to 28 mmHg, right atrial pressure from 8 to 11 mmHg. Volumes of the left ventricle also increase: the end systolic volume (VES) from 275 to 297 cm^3^, the end diastolic volume (VED) from 320 to 355 cm^3^. Concerning ventilation parameters we observe an increase of minute ventilation from 9.2 to 32.9 l/min and an increase of central arteriovenous oxygen difference from 7.0 to 13.1 ml/dl.

**Fig 4:**
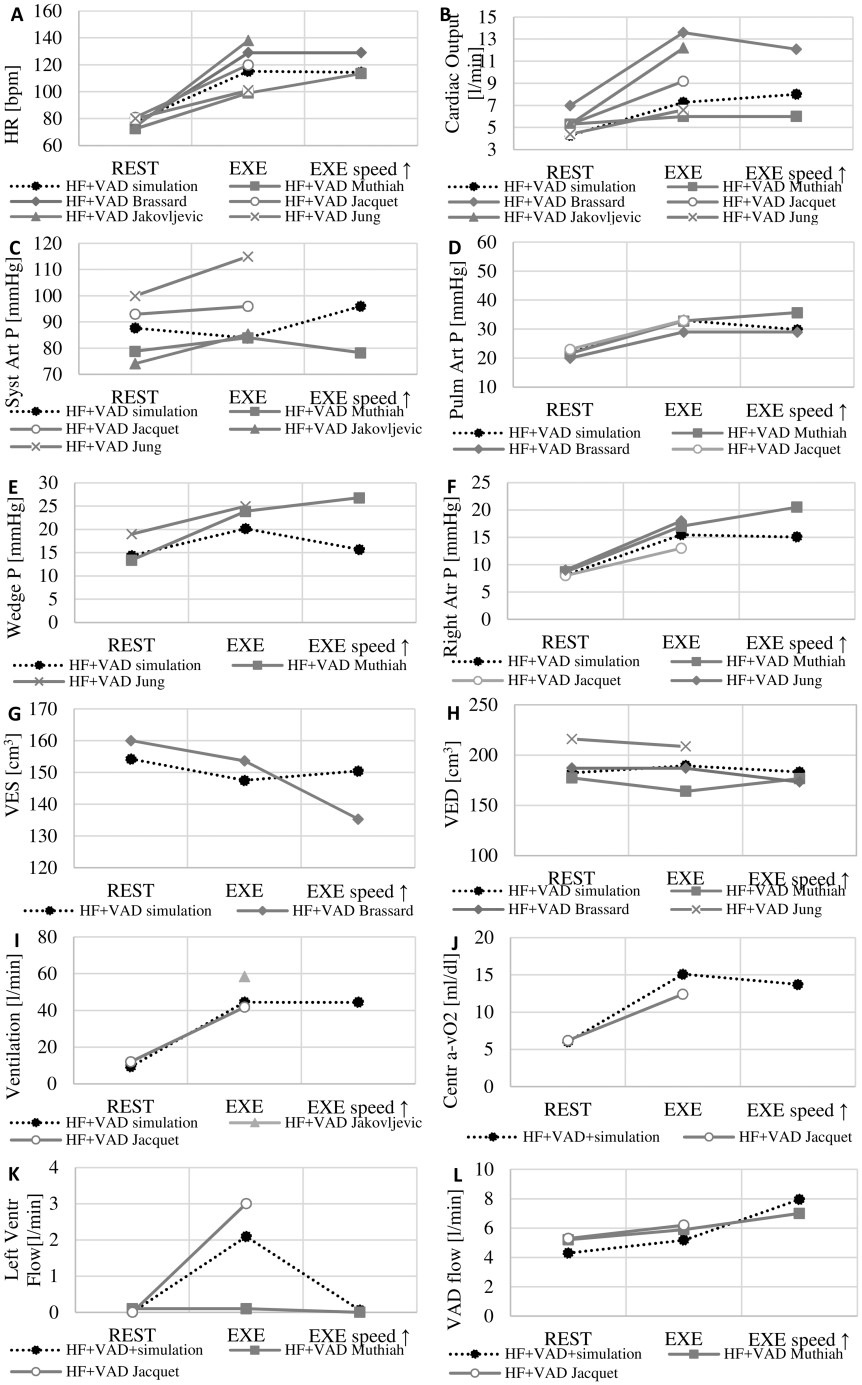
Comparison between literature and simulation studies at rest and at peak exercise in HF with a VAD. **Simulated** data refer to rest condition with a VAD run at 9500 rpm (REST), at peak exercise with a VAD at 9500 rpm (EXE) and at peak exercise with a VAD at 12000 rpm (EXE speed ↑). Data refer to: heart rate (A), cardiac output (B), aortic pressure (C), pulmonary arterial pressure (D), pulmonary capillary wedge pressure (E), right atrial pressure (F), left ventricular end systolic volume (G) and end diastolic volume (H), ventilation (I), central arteriovenous oxygen difference (J), left ventricular flow (K), VAD flow (L).

In Fig 4 we report the comparison between literature and simulation data concerning the HF+VAD. In this case simulation results refer to 3 conditions: at rest with HeartMate II running at 9500 rpm, at peak exercise with HeartMate II running at 9500 rpm and at peak exercise with HeartMate II running at 12000 rpm. Literature data refer to rest condition at baseline VAD speed, exercise condition at baseline VAD speed and exercise condition at increased VAD speed.

Parameters change in order as it follows: *HR* from 78 to 115 and 114 bpm, total cardiac output from 4.3 to 7.3 and 8.0 l/min, mean systemic arterial pressure from 88 to 84 and 96 mmHg, mean pulmonary arterial pressure from 22 to 33 and 30 mmHg, wedge pressure from 14 to 20 and 16 mmHg, right atrial pressure from 8 to 15 and 15 mmHg. Left ventricular end systolic volume changes from 154 to 147 and 150 cm^3^ and the end diastolic volume from 182 to 189 and 183 cm^3^. Minute ventilation increases from 9.2 to 44 and 44 l/min and central arteriovenous oxygen difference increases from 6.0 to 15.1 and 13.7 ml/dl. Left ventricular flow goes from 0 to 2.1 and 0 l/min, VAD flow changes from 4.3 to 5.2 and 8.0 l/min.

**Fig 5:**
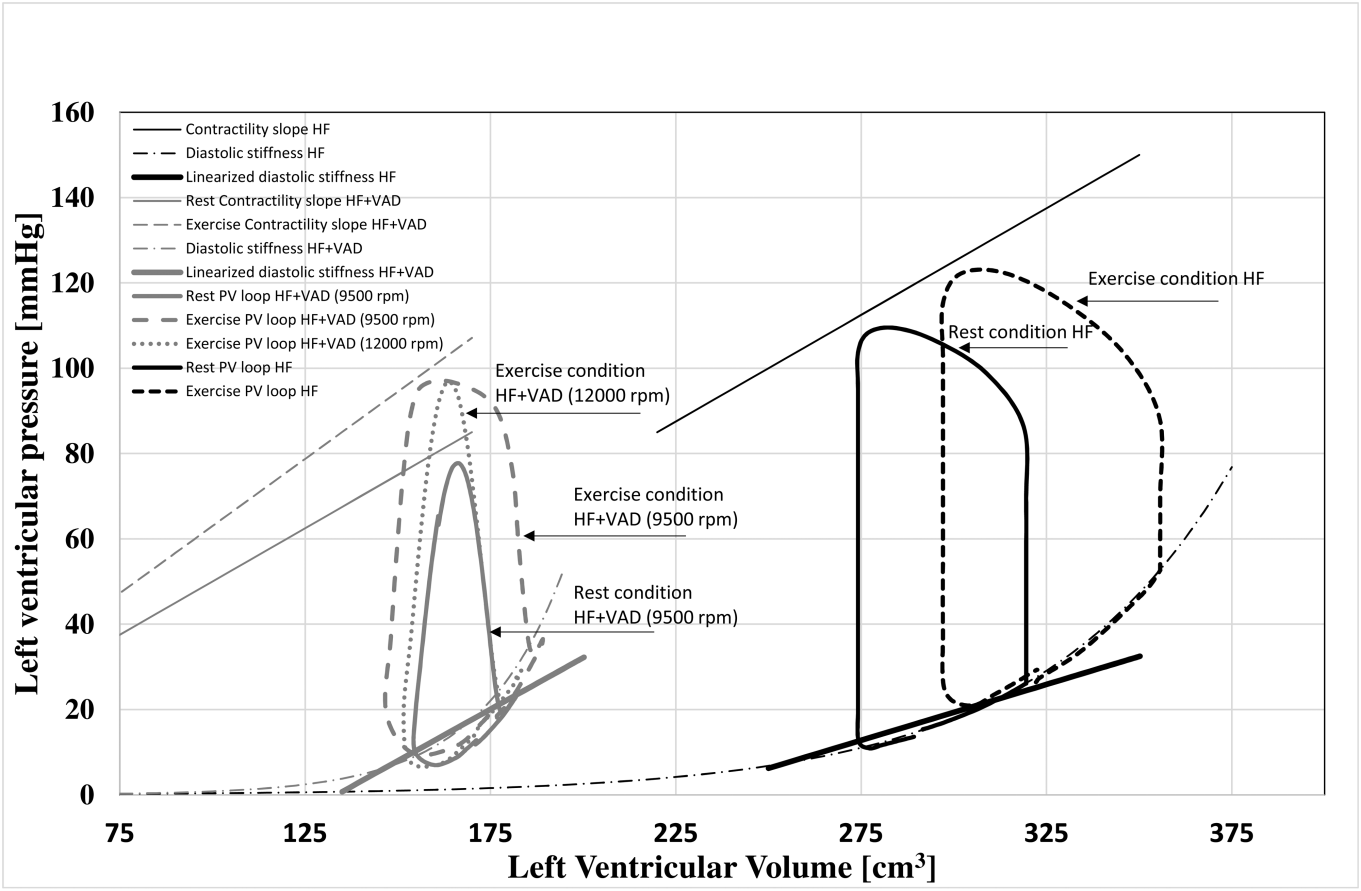
Left ventricular pressure-volume loops. Black waveforms: simulation of the left ventricular pressure-volume loops at rest and at peak exercise in heart failure condition with relative contractility and diastolic stiffness lines. Grey waveforms: simulation of the left ventricular pressure-volume loops at rest with a VAD run at 9500 rpm, at peak exercise with a VAD run at 9500 rpm and at 12000 rpm with relative contractility and diastolic stiffness lines.

In Fig 5 we report the simulations of the left ventricular pressure-volume loops in HF and in HF+VAD conditions. In the HF condition exercise moves the pressure-volume loops towards the right side of the plane and makes them wider (stroke volume increases from 45 to 58 cm^3^). In the HF+VAD condition ventricular volumes are in general narrower thanks to the unloading effect of the VAD. At rest condition, the left ventricle does not eject and its peak pressure is below the systemic arterial pressure. During exercise, Elvs increases and preload also increases, these two conditions make the left ventricle ejecting again. When the speed is increased to 12000 rpm, the VAD goes back to full support again and the left ventricle stops ejecting.

In Fig 5 we also plotted the diastolic stiffness of the HF and HF+VAD ventricles, according to Eq 2. In addition, a linearization of the diastolic function is represented around the working point of the ventricle at rest. The linearization results into a *dPlv/dVlv* slope of 0.26 mmHg/cm^3^ for HF and of 0.48 mmHg/cm^3^ for HF+VAD.

## Discussion

### HF condition

Exercise is a particular condition in which the heart and the circulation adapt to accommodate a higher cardiac output to better oxygenate peripheral regions. Venous return increases and the natural reaction of a healthy heart is to increase contractility and heart rate so that the end-diastolic ventricular volumes stay more or less the same as rest values.

In a failing heart, the increase of contractility is not so extensive or even absent so the myocardium resort to using the Frank Starling mechanism the ventricle increases its end diastolic volume in order to eject a larger stroke volume [20,21].

In case of a dilated heart, as we simulated in the present work, we observed an increase of both end-diastolic and end-systolic volumes (see Figs 3-G, 3-H and 5). Such a trend was also observed by [16] although in the upright position [22] the increase in end-diastolic volume is limited. Probably the hydrostatic pressure plays an important role on venous return so that different results could be observed weather the patient is cycling in upright or supine position. In the present model the effect of gravity was not considered, therefore our simulations better reflect supine exercise.

Concerning the capillary wedge pressure, previous studies [20,21] showed that it increases substantially in HF patients, as also observed from our simulations (see Fig 3-E). This phenomenon seems to be the base of ventricular filling during exercise. In a healthy heart the elastic recoil mechanism reduces ventricular pressure in early diastole, so that blood is naturally “sucked” from the atrium to the ventricular chamber [21]. In a HF ventricle, the elastic recoil mechanism is nearly absent but the elevation of atrial filling pressure compensates for this, assuring a sustained pressure drop across the mitral valve and therefore adequate filling during the shortened diastole while exercising [20]. The increase of the wedge pressure is associated also by an increase of the pulmonary pressure, as can be observed in Fig 3-D.

Systemic arterial pressure slightly increases in our simulator during exercise (Fig 3-C). This is in agreement with [3,16,17,18,22], although other works have shown the possibility of hypotension phenomena in HF subjects during exercise with reduced peripheral blood flow [23,24].

Some comments need to be addressed with respect to HR: according to our simulations HR at peak exercise is less than 80% of the expected value (considered as the threshold for chronotropic incompetence). Chronotropic incompetence is recognized to be one of the most important indicators of exercise limitation in HF and a critical variable for patients’ performance [25]. The simulated left ventricular ejection fraction is 14% at rest and 16% at exercise, corresponding to a strongly impaired ventricular function. The inadequate HR, together with a limited improvement of ventricular contractility, results in an insufficient increase of cardiac output. In our simulations cardiac output increases from 3.7 to 6.5 l/min, a value much lower than the cardiac output observed at peak exercise in healthy conditions (11.3 l/min according to Sullivan *et al*. [26]). The reduced cardiac output results into a lower amount of oxygen delivered to the tissues and as final result it induces a steep increase of central arteriovenous oxygen difference (see Fig 3-J).

Finally, according to results in Fig 3-I, ventilation (*Ve*) increases up to 32.9 l/min for a carbon dioxide removal (*VCO*_*2*_) of 1022 ml/ min. This corresponds to a *Ve/VCO*_*2*_ of 32 ml/ml, a value higher than the physiological threshold of 30 ml/ml and associated with ventilation inefficiency [25].

### HF+VAD condition

In the previous paragraph we provided a descriptive overview of exercise impairments in a HF condition and validated the simulator with data from literature. In our opinion this is a necessary step before reproducing VAD pathophysiology since, as we already stated, VAD and HF conditions share similar cardiovascular and ventilation impairments during exercise [4].

By comparing Figs 3-D, 3-E, 3-G, 3-H, 4-D, 4-E, 4-G and 4-H we observe that the HF+VAD at rest condition is characterized by a lower wedge pressure, lower pulmonary pressure and ventricular volumes and a higher cardiac output than HF. This reflects the beneficial effect of the VAD that drains blood from the left ventricle and pumps it into the systemic circulation.

But during exercise the beneficial effects of the VAD are less evident: the VAD is not capable to refrain wedge pressure and the pulmonary pressure to increase and cardiac output augmentation is just comparable to the one observed in HF (+2.8 l/min in HF and +3.0 l/min in HF+VAD).

The increase of wedge pressure was already shown by Hayward et al [27], but the mechanism behind it has not been clarified so far. According to our simulator, wedge pressure increases because of the diastolic stiffness of the ventricle. More specifically:

- The ventricle with a VAD has stiffer elastic properties than failed unassisted ventricles. This means that for the same ventricular volume, wedge pressure is higher in HF+VAD than in HF.
- The native ventricle under VAD works in a very non-linear portion of the diastolic stiffness. The linearization of the *dPlv/dVlv* relationship, shown in Fig 5, evidences a steeper slope for HF+VAD than for HF. This means that for a comparable increase of ventricular volumes during exercise, wedge pressure would increase more in HF+VAD than in HF condition. According to Fig 5 end diastolic volume does not increase substantially (from 182 to 189 cm^3^) in HF+VAD. These results are in agreement with the echo measurements reported in [2,5,13] showing that the diameter of the left ventricle stays rather constant during exercise. But since the ventricle works in the nonlinear portion of the diastolic stiffness, even a small increase of volume results in a substantial increase of wedge pressure (from 14 to 20 mmHg).

The two observations above are the result of the so called “reverse-remodeling” operated by the VAD. The hemodynamic unloading of VAD on the ventricle stops the vicious cycle of ventricular dysfunction and dilatation observed during heart failure [28]. Fig 2-B clearly shows that VAD contributes to increase the ventricular stiffness towards values that are closer to normal ones (Fig 2-B).

Concerning *CO*, the simulator shows that (see Fig 4-K and 4-L):

- At peak exercise CO increases of +3.0 l/min, a values just comparable to the one observed in HF condition and below the expected value in a healthy subject,
- CO increase is a combined result of the native ventricle and the VAD. From hydraulic point of view, in fact, the VAD and the left ventricle are two pumps working in parallel. Hence, the total cardiac output is the sum of both contributions. At rest condition the ventricle does not eject (we simulated a full support), but during exercise the ventricle provides a significant contribution to total cardiac output (2.1 l/min). The VAD is capable to increase its flow only by +0.9 l/min at exercise.

Given this limited increase of CO and the modest contribution of the VAD to it, we investigated if VAD speed modulation could provide some benefits in terms of hemodynamics during exercise. So far the influence of VAD speed increase was investigated in several studies, some reporting significant effects on hemodynamics [2,29] and some not [5,6]. Noor et al. [30] evidenced that patients with a poorer left ventricular function (ejection fraction <40%) are more sensitive to VAD speed changes during exercise. For our simulations, we opted for a left ventricular ejection fraction at rest of 15%, corresponding to the average condition of patients in our clinical center.

It is also important to remark that most of the previous studies investigated a relatively small increase of VAD speed and therefore the question arises whether a more important VAD speed change would have provided a better patient performance. In this study, thanks to the use of a simulator, we could investigate the effects of a VAD running at the maximal possible speed (12000 rpm for the HeartMate II).

By increasing the VAD speed we could obtain a higher total cardiac output and an increase of VAD flow compared to exercise at baseline speed (+0.7 l/min and +2.8 l/min, respectively). Basically, VAD speed modulation has a large effect on left ventricular unloading but affects CO only mildly.

VAD speed also decreases wedge pressure by -4.5 mmHg (see Fig 4-E), which seems to be in contradiction with Muthiah et al. [5] noticing even an increase of wedge pressure due to VAD speed modulation. This might be due to two different reasons: Muthiah et al. [5] performed a modest increase of VAD speed compared to this study with an increase of VAD flow of only 1.1 l/min, pump speed was increased after the patients were already exercising for 5 minutes.

The increase of VAD speed does not affect ventilation substantially but it reduces central arteriovenous oxygen difference of -1.4 ml/dl (Fig 4-I and 4-J). This means that a higher VAD speed can assure a better oxygenation of tissues with possible positive consequences in terms of reduced fatigue of active muscles.

To summarize, by combining the literature observations and our simulation results we could speculate that exercise in VAD patients is a very peculiar condition both during systole and diastole. The native ventricle becomes stiffer after the VAD implantation so that the wedge pressure rises even for small changes of ventricular volumes. One could also argue that the decrease in end-diastolic volume, operated by the VAD, is beneficent to decrease systolic wall tension, but is contra-productive by increasing diastolic chamber stiffness which becomes the major obstacle to increased filling and thus stroke volumes.

The VAD effect on ventricular stiffness, might not be accompanied by a significant improvement in the myocyte function and in the overall myocardial contractile strength [12]. Hence a failed heart is not capable to accommodate a higher CO the same way a healthy heart would do. A healthy ventricle is regulated by the Frank Starling mechanism and it is subject to a positive chronotropic and inotropic effects during exercise. All these regulatory mechanisms are impaired in a failed ventricle.

The VAD also shows an inadequate capability in improving its flow during exercise. Our simulations show that in a failed unassisted ventricle CO increases of +2.8 l/min (from 3.7 to 6.5 l/min). Similarly, in a failed assisted ventricle CO increases of +3.0 l/min (from 4.3 to 7.3 l/min), +2.1 l/min due to the left ventricle and the remaining +0.9 l/min due to the VAD. There results evidence that VAD is not capable to properly increase CO neither to unload the left ventricle during exercise.

By increasing VAD speed to the highest limit we were just able to re-establish a condition of full support again but total cardiac output reached a plateau of 8.0 l/min. This indicates that VAD speed modulation can produce a better unloading of the left ventricle, but it has a moderate effect on total cardiac output. Future studies should be conducted to investigate whether an even higher support, at present not available with the current VAD technology, could positively affect cardiac output or whether other factors like right ventricular function, peripheral resistance etc. would limit cardiac output anyway. In addition, it is worth to underline that we simulated competent heart valves. As future step, the role of aortic insufficiently, a rather common complications in VAD patients, should be investigated especially in the hypothesis of a VAD speed modulation.

## Conclusions

Observations of hemodynamic changes in patients with VAD during exercise were collected. A cardio-respiratory simulator was updated with these fragmented data allowing the most reliable simulation of exercise physiology of patient with heart failure and VAD. The simulator indicates that exercise capacity remains limited in VAD patients mainly because of an increased left ventricular stiffness and a non-optimal unloading of the left ventricle at peak exercise.

## Supporting information

S1 Table Simulator parameters. List of additional parameters used to characterize the simulator in HF and in VAD rest conditions.
(DOCX)
