# Supplementary material for: Exercise physiology with a left ventricular assist device: Analysis of heart-pump interaction with a computational simulator
Source: PLoS One. 2017 Jul 24;12(7):e0181879. doi: 10.1371/journal.pone.0181879 (PMC5524292; doi:10.1371/journal.pone.0181879)
Supplement: S1 Table — List of additional parameters used to characterize the simulator in HF and in VAD rest conditions. (DOCX) [file pone.0181879.s001.docx]

# Supporting information

In this section we report the improvements implemented in the model. All parameters values, relative to this section, are reported in S1 Table 1.

**S1 Table 1. Simulator parameters**

| **Symbol** | **Parameter** | **Unit** | **HF** | **HF+VAD** |
| --- | --- | --- | --- | --- |
| **A**  **B**  **C** | Parameters of VAD pressure-flow characteristics at 9500/12000 rpm | mmHg/(l/min)^2^  mmHg/(l/min)  mmHg | - | -0.33/ -0.88  -8.65/ -5.24  116/183 |
| ***C_0ub_*** | Upper body basal compliance | cm^3^/mmHg | 8 | 8 |
| ***C_0sp_*** | Splanchnic basal compliance | cm^3^/mmHg | 55 | 55 |
| ***C_0ll/_ C_0rl_*** | Left/right leg basal compliance | cm^3^/mmHg | 9.5/9.5 | 9.5/9.5 |
| ***C_0ivf_*** | Inferior vena cava basal compliance | cm^3^/mmHg | 25 | 25 |
| ***Elamax/Eramax*** | Left/right atrial maximal elastance | mmHg/cm^3^ | 0.25/0.25 | 0.25/0.25 |
| ***Elamin/Eramin*** | Left/right atrial minimal elastance | mmHg/cm^3^ | 0.1/0.1 | 0.1/0.1 |
| ***C_O2ubvRef_*** | Reference venous oxygen concentration in the upper body | ml O_2_/dl blood | 10 | 10 |
| ***C_O2kidvRef_*** | Reference venous oxygen concentration in the kidneys | ml O_2_/dl blood | 13.5 | 13.5 |
| ***C_O2spvRef_*** | Reference venous oxygen concentration in the splanchnic circulation | ml O_2_/dl blood | 11 | 11 |
| ***C_O2llvRef_/C_O2rlvRef_*** | Reference venous oxygen concentration in the left/right leg | ml O_2_/dl blood | 10 | 10 |
| ***Vla_0_/Vra_0_*** | Left/right atrial zero pressure volume | cm^3^ | 10/10 | 10/10 |
| ***ΔV_maxub_*** | Maximal upper body volume change | cm^3^ | 250 | 250 |
| ***ΔV_maxsp_*** | Maximal splanchnic volume change | cm^3^ | 1400 | 1400 |
| ***ΔV_maxll/rl_*** | Maximal left/right leg volume change | cm^3^ | 175/175 | 175/175 |
| ***ΔV_maxivc_*** | Maximal inferior vena cava volume change | cm^3^ | 578 | 578 |
| **T_AE0_-T_AB0_** | Baseline duration of atrial contraction | ms | 150 | 150 |
| **TD_0_** | Baseline duration of diastole | ms | 611 | 611 |

List of additional parameters used to characterize the simulator in HF and in VAD rest conditions.

## Atrial contraction

A model of atrial contraction was implemented in the simulator according to [1]:

$$ac\left( t \right)=\left\{ \begin{aligned} 0, 0 \leq t < T_{AB} \\ \frac{1-cos\left( \frac{t-T_{AB}}{T_{AE}-T_{AB}}\cdot2\pi\right)}{2}, T_{AB} \leq t < T_{AE} \\ 0, t\geq T_{AE} \end{aligned} \right.$$

(S1. 1)

where *ac* is the atrial contraction function, *T_AB_* (*T_AE_*) is the beginning (ending) time of atrial contraction.

The atrial chamber is represented by the following equation:

*Pla(t)=[Elamin+(Elamax-Elamin)·ac(t)] ·(Vla-Vla_0_)+Pintr(t)*

(S1. 2)

Where *Pla* is the left atrial pressure, *Vla* is the left atrial volume, *Vla_0_* is the left atrial volume at zero pressure, *Elamax* (*Elamin*) is the maximum and minimum value of elastance, *Pintr* is the intrathoracic pressure. For the right atrium a similar equation was implemented.

The ventricular contraction function *vc* was also taken from [1]:

$$vc\left( t \right)=\left\{ \begin{aligned} \frac{1-cos\left( \frac{t}{T_{VH}}\pi\right)}{2}, 0 \leq t < T_{VH} \\ \frac{1+cos\left( \frac{t-T_{VH}}{T_{VE}-T_{VH}}\cdot2\pi\right)}{2}, T_{VH} \leq t < T_{VE} \\ 0, t\geq T_{VE} \end{aligned} \right.$$

(S1. 3)

Where *T_VH_* (*T_VE_*) is the half (ending) time of ventricular contraction.

The systole/diastole duration ratio (T*S/TD*) varies according to heart rate (*HR*) as it follows:

*TS/TD=0.0001·HR^2^-0,017·HR+1.297*

(S1. 4)

The duration of atrial contraction *T_AE_-T_AB_* is then calculated as it follows:

$$T_{AE-}T_{AB}=TD\cdot\left( T_{AE0-}T_{AB0} \right)/{TD}_{0}$$

(S1. 5)

Where *T_AE0_-T_AB0_* is the duration of atrial contraction and *TD_0_* is the duration of diastole at HR of 60 bpm.

The duration of ventricular contraction corresponds to *TS* so that *T_VE_*=*TS* and *T_VH_*=*TS*/2.

## Non-linear vascular compliance

A model for non-linear venous compliances was implemented in the upper body, lower limbs, splanchnic and inferior vena cava regions. The model was taken from [2]:

$$\Delta V(t)=\frac{2\cdot{\Delta V}_{maxi}}{\pi}arctan\left( \frac{\pi\cdot C_{0i}}{2\cdot{\Delta V}_{maxi}}\cdot\Delta P_{trans}(t) \right)$$

(S1. 6)

Where *ΔV* (*ΔV_maxi_*) is the change (maximal change) of the i^th^ compartment volume, *ΔP_trans_* is the transmural pressure and *C_0i_* is the compliance of the i^th^ compartment at the basal transmural pressure.

*Inverse relationship between pulmonary arterial compliance and resistance*

The relationship between pulmonary arterial compliance and resistance (*Cap*, *Rap*) and wedge pulmonary capillary wedge pressure (*Pwedge*) was represented as it follows:

$$Cap=\frac{0.711-0.0163\cdot\left( Pwedge-11 \right)}{{0.051-0.0006\cdot\left( Pwedge-11 \right)+Rap}}$$

(S1. 7)

The equation was obtained by fitting the data from [3].

## VAD model

The VAD was modelled as a flow generator according to the pressure-flow characteristics of HeartMate II (Thoratec Corporation) [4]:

*∆P(t)=A·Q^2^_VAD_(t)+B·Q_VAD_(t)+C*

(S1. 8)

Where *ΔP* is the pressure drop across the VAD, *Q_VAD_* is the VAD flow and *A*, *B* and *C* are constant parameters that depend on VAD speed. The VAD model was connected between the left ventricle and the aorta. A schematic overview of the VAD model connection to the cardiovascular system is reported in Fig 1.

# References

1. Korakianitis T, Shi Y. A concentrated parameter model for the human cardiovascular system including heart valve dynamics and atrioventricular interaction. Medical Engineering & Physics. 2006;28(7), 613–28.
2. Heldt T, Shim EB, Kamm RD, Mark RG. Computational modeling of cardiovascular response to orthostatic stress. J Appl Physiol. 2002;92(3), 1239–54.
3. Tedford RJ, Hassoun PM, Mathai SC, Girgis RE, Russell SD, Thiemann DR et al. Pulmonary Capillary Wedge Pressure Augments Right Ventricular Pulsatile Loading. Circulation. 2012;125(2), 289–97.
4. Sunagawa G, Byram N, Karimov JH, Horvath DJ, Moazami N, Starling RC et al. In vitro hemodynamic characterization of HeartMate II at 6000 rpm: Implications for weaning and recovery. J Thorac Cardiovasc Surg. 2015;150(2), 343-8.
